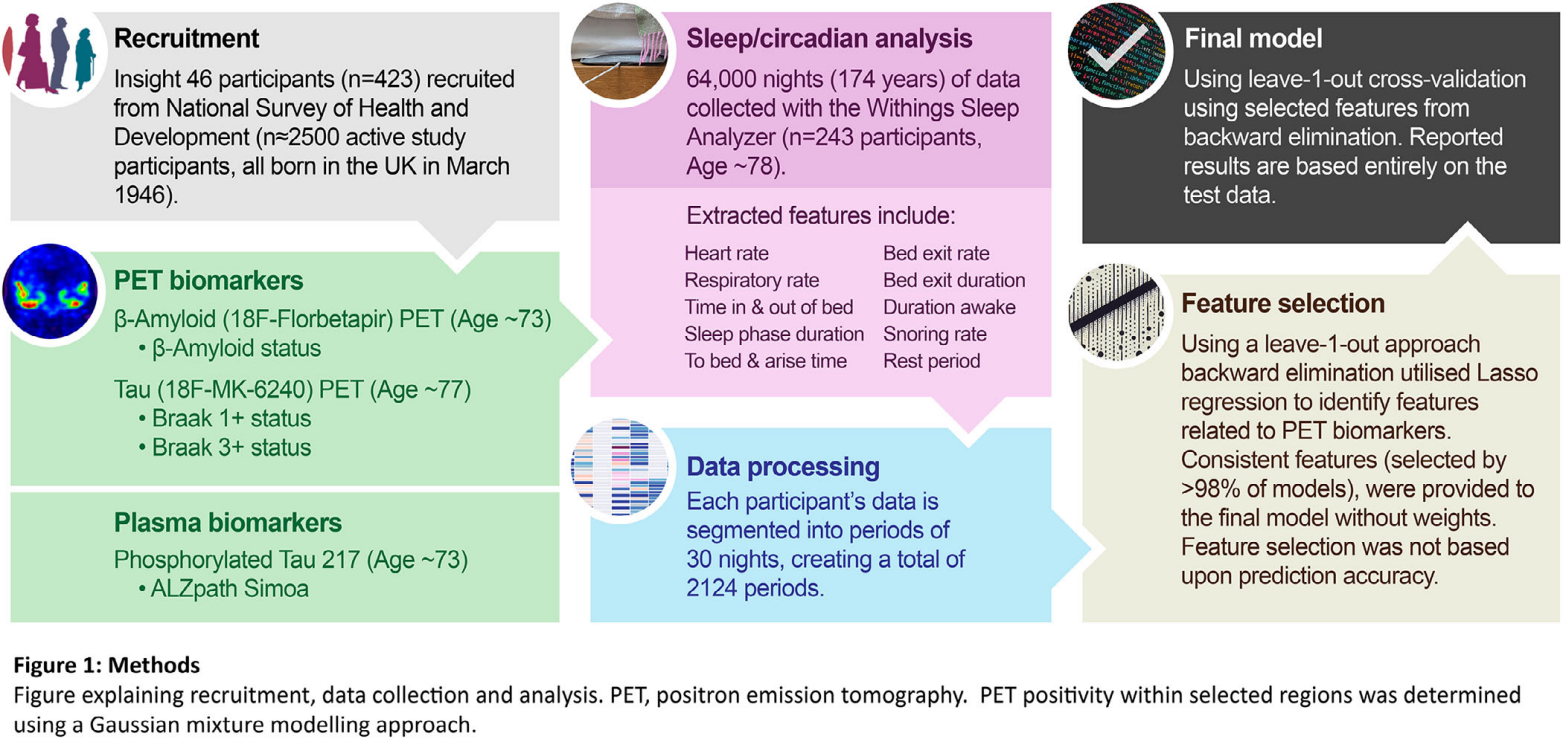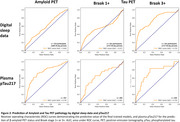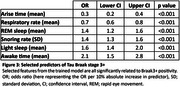# Remote detection of Alzheimer's disease pathology using a digital sleep and circadian biomarker: an InSleep46 study

**DOI:** 10.1002/alz70863_110651

**Published:** 2025-12-23

**Authors:** Josh King‐Robson, Eyal Soreq, Molly R E Cartlidge, Matthew Harrison, Heidi Murray‐Smith, Lina Aimola, Marie Poole, Ríona Mc Ardle, Ashvini Keshavan, David M Cash, William Coath, Louise Robinson, David J Sharp, Jonathan M Schott

**Affiliations:** ^1^ Dementia Research Centre, UCL Queen Square Institute of Neurology, University College London, London UK; ^2^ Department of Brain Sciences, Imperial College London, London UK; ^3^ UK Dementia Research Institute Centre for Care Research & Technology, Imperial College London and University of Surrey, London UK; ^4^ Sorpol Consultancy, Ashdod Israel; ^5^ Population Health Sciences Institute, Newcastle University, Newcastle upon Tyne UK; ^6^ NIHR Newcastle Biomedical Research Centre, Newcastle University, Newcastle upon Tyne, Tyne and Wear UK; ^7^ Translational and Clinical Research Institute, Newcastle University, Newcastle upon Tyne, Tyne and Wear UK

## Abstract

**Background:**

Sleep and circadian disruption are associated with increased dementia risk. Digital sleep biomarkers may provide an ecologically valid and low‐burden means of remote population‐level screening for incipient dementia. We explored the feasibility and predictive value of a digital sleep biomarker, developed from data collected using the Withings Sleep Analyzer (WSA), a ballistocardiographic under‐mattress pressure sensor which collects sleep and physiological data unobtrusively, to detect Alzheimer‐related biomarkers in a presymptomatic cohort.

**Method:**

Participants from the Insight 46 study (all born in March 1946) underwent serial assessment, including plasma phosphorylated tau (pTau)217 ALZpath and 18F‐Florbetapir β‐amyloid PET at age ∼73 and 18F‐MK‐6240 Tau PET at age ∼77. Amyloid status (‐/+) and Tau Braak staging (‐/Braak1+/Braak3+) were derived using automated pipelines.

The WSA was deployed at age ∼78, installed under participants’ mattresses by the study participant/family. Continuous sleep, circadian, and physiological parameters were collected. A leave‐one‐out cross validation approach was employed to develop models predicting PET status after feature selection (Figure 1). Results were compared to plasma pTau217.

**Result:**

*n* = 161 had both WSA and Tau PET data (12.4% Braak1+, 6.2% Braak3+); *n* = 153 participants also had β‐amyloid PET (25% β‐amyloid+ at Centiloid>=12). In total we collected 63,720 nights (174 years) of sleep data, corresponding to a mean±SD of 239.8±108.7 nights/participant (age at collection 78.3±0.2 yrs; 49% female). *n* = 404 had plasma pTau217.

A final trained model identified asymptomatic individuals with Braak3+ tau pathology with area under the receiver operating characteristic curve (AUROC)=0.75; comparable to plasma pTau217 (Figure 2) after iterative feature selection (Figure 3). Trained models were less effective at identifying earlier pathological stages (Tau Braak1+, β‐amyloid+).

**Conclusion:**

Deploying a remote sleep and circadian monitoring device in a countrywide population‐based cohort in their late 70s is feasible. A model based on iterative feature selection was able to identify individuals with significant Tau (Braak3+) pathology with AUROC similar to plasma pTau217. This provides proof‐of‐concept that digital sleep biomarkers may be useful in identifying individuals at high risk of developing clinical AD. Work is underway to refine the model further, replicate these results in other cohorts, and identify the shortest duration of recording required for robust prediction.